# Changes in the Editorial Staff of This Journal

**Published:** 1879-07

**Authors:** 


### Editorial.

### ARTICLE XIV.

### CHANGES IN THE EDITORIAL STAFF OF THIS JOURNAL.

With the present number begins the thirty-ninth volume of the CHICAGO MEDICAL JOURNAL AND EXAMINER. It will be seen that a change has been effected in the editorial staff, one of those changes which were contemplated when this publication became the property of the Chicago Medical Press Association. It was then designed to secure such flexibility in the editorial management, that the journal might, as far as possible, remain a representative of the medical profession of Chicago, and that changes in the corps of editors might not affect either its character or influence.

We are called upon to write at once a valedictory and a salutatory. Dr. F. C. Hotz and Dr. E. F. Ingals retire from connection with the editorial management, voluntarily and after much effort upon our part to secure their services for the future. Both of these gentlemen are burdened with duties which will hereafter prevent their devoting time to the labor involved in editing and publishing a work of this character. To both of them, the readers and subscribers of this journal are greatly indebted, for their excellent and efficient co-operation. It is unnecessary to say that they have also largely contributed to that spirit of harmony and good feeling which has from the first characterized our journalistic labors with them. They resign to carry with them the regrets and best wishes of their confrères in the editorial staff.

One vacancy, thus created, is filled by Dr. N. S. Davis, Dean of the Faculty of the Chicago Medical College, a gentleman who certainly needs no introduction to the medical profession of this country or to our colleagues of the medical press. Veteran editor and author, he comes to us as one who returns to a familiar field. Dr. Davis has been in turn editor of each of the two periodicals whose union gave birth to the present JOURNAL AND EXAMINER. He will be welcomed to his new position by his many friends throughout the country, and his associates congratulate themselves, as well as the readers of these pages, upon the strength which is thus added to their organization.

Dr. D. R. Brower, one of the faculty of the Woman's hospital Medical College of Chicago, succeeds to the charge of the publication department of this journal. Dr. Brower has been for some time associated in the work of this department and will bring to it the fruit of his extended experience. There will be no change in the address of the publication in consequence of this transfer, as all communications should continue to be sent, as heretofore, to No. 188 S. Clark St., Chicago, where are the rooms of the Press Association.

It should be added that the prospects of this periodical were never brighter than at present. We commence the present volume with a full subscription-list, free from all indebtedness, and with a handsome balance in bank. The conduct of this journal has been such in the past as to demonstrate the fact that a thoroughly independent and non-partisan sheet, whose sole aim is the advancement of the interests of science and scientific men the world over, not only deserves the support of such men, but receives it in such manner as to achieve financial success. It only remains for us to say that no pains will be spared in the future to render these pages valuable to the student, the practitioner, the author and the teacher of medicine.

---

A GERMAN physician wanted for a thriving Nebraska city. A most excellent opportunity for a German physician, well educated and of good habits. His statements must be reliably substantiated. Address for particulars Dr. A. S. V. Mansfelde, Secretary of Nebraska State Medical Society, Ashland, Nebraska.

